# New Horizons for Personalised Treatment in Gastroesophageal Cancer

**DOI:** 10.3390/jcm11020311

**Published:** 2022-01-09

**Authors:** Massimiliano Salati, Andrea Spallanzani

**Affiliations:** 1 Division of Oncology, Department of Oncology and Hematology, University Hospital of Modena, 41124 Modena, Italy; spallanzani.andrea@aou.mo.it; 2 PhD Program Clinical and Experimental Medicine, University of Modena and Reggio Emilia, 41121 Modena, Italy

Gastric and gastroesophageal junction adenocarcinoma (GEA) is still responsible for a huge health burden worldwide, being the second most common cause of cancer-related death globally [1]. Even though GEA is not ranked in the top-five malignancy in developed countries, its incidence is increasing among younger individuals, particularly at the proximal anatomical subsite [2].

The recent years have witnessed unprecedented advances in the field of surgical techniques, loco-regional procedures and systemic therapies that have enabled incremental yet steady improvements in patients’ outcomes and have made the multidisciplinary approach an unavoidable need in GEA. In parallel, the availability of high-throughput technologies has deepened the molecular understanding of the disease, unveiling considerable biological heterogeneity and vulnerabilities.

In potentially resectable GEA, particularly in Western countries, taxane-based triplet chemotherapy has been established as the new reference perioperative regimen, which provides a 10% increase in curability in the growing proportion of patients who are given upfront systemic treatment versus the previous anthracyclines-based standard of care [3]. In this setting, the MSI testing at diagnosis is increasingly performing: MSI-h tumours had a better prognosis with surgery alone and a recent meta-analysis reported detrimental survival with platinum-fluoropyrimidine based perioperative chemotherapy [4], suggesting in some centres, an upfront surgery approach, while others are investigating chemo-free immunotherapy-based approaches, such as that pursued in the currently ongoing INFINITY trial [5].

In metastatic GEA, after years of stagnation and a plateau for standard cytotoxic polychemotherapy, the molecular segmentation that emerged from the TCGA project has provided a roadmap for personalised treatments [6]. In HER2-negative GEA with PD-L1 CPS ≥ 5, the addition of the anti-PD1 nivolumab to first-line chemotherapy provided practice-changing results in the phase III Check-Mate-649 trial [7]. There is now convincing evidence also for GEA that MSI-h metastatic tumours derived greater benefit from immune checkpoint blockade than standard therapy [8], while this finding needs prospective confirmation for the subset of TMB-H and EBV-positive tumours.

Among the molecular subgroup of HER2-positive GEA, a renewed enthusiasm surrounds novel targeted agents of which trastuzumab deruxtecan is the most promising at this point [9]. This antibody–drug conjugate against HER2 produced a remarkable improvement in response rate (51% vs. 14%) and overall survival (12.5 vs. 8.4 months) when compared to chemotherapy in heavily pretreated patients.

Regarding novel tools for precision medicine, blood-based ctDNA analysis (referred to as liquid biopsy) is showing potential transformative applications in GEA, including the detection of minimal residual disease after curative treatment, the capture of resistance mechanisms and the identification of new targets in GEA [10,11,12]. Moreover, advances in preclinical cancer models have led to the establishment of patient-derived organoids as rapid, reliable and cost-effective tools to run high-throughput 3D drug screening, study drug resistance and, more interestingly, model treatment response to systemic therapies [13].

This Special Issue aims to discuss open questions, highlight major innovations and depict future perspectives in the multidisciplinary management of gastroesophageal cancer, bridging together major specialties committed to the personalisation and improvement of GEA patient care.

## References

[1] Sung H., Ferlay J., Siegel R.L., Laversanne M., Soerjomataram I., Jemal A., Bray F. (2021). Global Cancer Statistics 2020: GLOBOCAN Estimated Cancer Incidence, Mortality and Prevalence Worldwide for 36 cancers in 185 countries. CA Cancer J. Clin..

[2] Anderson W.F., Camargo M.C., Fraumeni J.F., Correa P., Rosenberg P.S., Rabkin C.S. (2010). Age-specific trends in incidence of non-cardia gastric cancer in US adults. JAMA.

[3] Al-Batran S.-E., Homann N., Pauligk C., Goetze T.O., Meiler J., Kasper S., Kopp H.-G., Mayer F., Haag G.M., Luley K. (2019). Perioperative chemotherapy with fluorouracil plus leucovorin, oxaliplatin, and docetaxel versus fluorouracil or capecitabine plus cisplatin and epirubicin for locally advanced, resectable gastric or gastro-oesophageal junction adenocarcinoma (FLOT4): A randomised, phase 2/3 trial. Lancet.

[4] Pietrantonio F., Miceli R., Raimondi A., Kim Y.W., Kang W.K., Langley R.E., Choi Y.Y., Kim K.-M., Nankivell M.G., Morano F. (2019). Individual Patient Data Meta-Analysis of the Value of Microsatellite Instability as a Biomarker in Gastric Cancer. J. Clin. Oncol..

[5] Raimondi A., Palermo F., Prisciandaro M., Aglietta M., Antonuzzo L., Aprile G., Berardi R., Cardellino G.G., de Manzoni G., de Vita F. (2021). TremelImumab and Durvalumab Combination for the Non-OperatIve Man-agement (NOM) of Microsatellite InstabiliTY (MSI)-High Resectable Gastric or Gastroesophageal Junction Cancer: The Multicentre, Single-Arm, Multi-Cohort, Phase II INFINITY Study. Cancers.

[6] Salati M., Orsi G., Smyth E., Beretta G., de Vita F., di Bartolomeo M., Fanotto V., Lonardi S., Morano F., Pietrantonio F. (2019). Gastric cancer: Translating novels concepts into clinical practice. Cancer Treat. Rev..

[7] Janjigian Y.Y., Shitara K., Moehler M., Garrido M., Salman P., Shen L., Wyrwicz L., Yamaguchi K., Skoczylas T., Bragagnoli A.C. (2021). First-line nivolumab plus chemotherapy versus chemotherapy alone for advanced gastric, gastro-oesophageal junction, and oesophageal adenocarcinoma (CheckMate 649): A randomised, open-label, phase 3 trial. Lancet.

[8] Pietrantonio F., Randon G., di Bartolomeo M., Luciani A., Chao J., Smyth E., Petrelli F. (2021). Predictive role of microsatellite instability for PD-1 blockade in patients with advanced gastric cancer: A meta-analysis of randomized clinical trials. ESMO Open.

[9] Shitara K., Bang Y.-J., Iwasa S., Sugimoto N., Ryu M.-H., Sakai D., Chung H.-C., Kawakami H., Yabusaki H., Lee J. (2020). Trastuzumab Deruxtecan in Previously Treated HER2-Positive Gastric Cancer. N. Engl. J. Med..

[10] Leal A., van Grieken N.C.T., Palsgrove D.N., Phallen J., Medina J.E., Hruban C., Broeckaert M.A.M., Anagnostou V., Adleff V., Bruhm D.C. (2020). White blood cell and cell-free DNA analyses for detection of residual disease in gastric cancer. Nat. Commun..

[11] Maron S.B., Chase L.M., Lomnicki S., Kochanny S., Moore K.L., Joshi S.S., Landron S., Johnson J., Kiedrowski L.A., Nagy R.J. (2019). Circulating Tumor DNA Sequencing Analysis of Gastroesophageal Adenocarcinoma. Clin. Cancer Res..

[12] Salati M., Venetis K., Fassan M., Malapelle U., Pagni F., Sajjadi E., Fusco N., Ghidini M. (2021). ctDNA analysis in the personalized clinical management of gastroesophageal adeno-carcinoma: Turning hope into reality. Future Oncol..

[13] Seidlitz T., Merker S.R., Rothe A., Zakrzewski F., von Neubeck C., Grützmann K., Sommer U., Schweitzer C., Schölch S., Uhlemann H. (2019). Human gastric cancer modelling using organoids. Gut.

